# Transcriptional profiling of reproductive development, lipid storage and molting throughout the last juvenile stage of the marine copepod *Calanus finmarchicus*

**DOI:** 10.1186/s12983-014-0091-8

**Published:** 2014-12-16

**Authors:** Ann M Tarrant, Mark F Baumgartner, Bjørn Henrik Hansen, Dag Altin, Trond Nordtug, Anders J Olsen

**Affiliations:** Biology Department, Woods Hole Oceanographic Institution, 45 Water Street, Woods Hole, MA 02543 USA; SINTEF Materials and Chemistry, Environmental Technology, N-7465 Trondheim, Norway; BioTrix, N-7022 Trondheim, Norway; Department of Biology, Norwegian University of Science and Technology, N-7491 Trondheim, Norway

**Keywords:** Arthropod, Crustacean, Gene expression, Molt cycle, Transcriptomics

## Abstract

**Introduction:**

*Calanus finmarchicus*, a highly abundant copepod that is an important primary consumer in North Atlantic ecosystems, has a flexible life history in which copepods in the last juvenile developmental stage (fifth copepodid, C5) may either delay maturation and enter diapause or molt directly into adults. The factors that regulate this developmental plasticity are poorly understood, and few tools have been developed to assess the physiological condition of individual copepods.

**Results:**

We sampled a cultured population of *C. finmarchicus* copepods daily throughout the C5 stage and assessed molt stage progression, gonad development and lipid storage. We used high-throughput sequencing to identify genes that were differentially expressed during progression through the molt stage and then used qPCR to profile daily expression of individual genes. Based on expression profiles of twelve genes, samples were statistically clustered into three groups: (1) an early period occurring prior to separation of the cuticle from the epidermis (apolysis) when expression of genes associated with lipid synthesis and transport (FABP and ELOV) and two nuclear receptors (ERR and HR78) was highest, (2) a middle period of rapid change in both gene expression and physiological condition, including local minima and maxima in several nuclear receptors (FTZ-F1, HR38b, and EcR), and (3) a late period when gonads were differentiated and expression of genes associated with molting (Torso-like, HR38a) peaked. The ratio of Torso-like to HR38b strongly differentiated the early and late groups.

**Conclusions:**

This study provides the first dynamic profiles of gene expression anchored with morphological markers of lipid accumulation, development and gonad maturation throughout a copepod molt cycle. Transcriptomic profiling revealed significant changes over the molt cycle in genes with presumed roles in lipid synthesis, molt regulation and gonad development, suggestive of a coupling of these processes in *Calanus finmarchicus*. Finally, we identified gene expression profiles that strongly differentiate between early and late development within the C5 copepodid stage. We anticipate that these findings and continued development of robust gene expression biomarkers that distinguish between diapause preparation and continuous development will ultimately enable novel studies of the intrinsic and extrinsic factors that govern diapause initiation in *Calanus finmarchicus*.

**Electronic supplementary material:**

The online version of this article (doi:10.1186/s12983-014-0091-8) contains supplementary material, which is available to authorized users.

## Introduction

Copepods of the family Calanidae, including the *Calanus* and *Neocalanus* genera, are vital components of temperate, subarctic, and arctic ecosystems that transfer energy directly from phytoplankton to very high trophic levels (e.g., fish, sea birds, and some marine mammals). The life history of these copepods typically includes diapause, a period of arrested development, dormancy and fasting that can last many months [1-3]. The well-studied temperate and subarctic copepod, *Calanus finmarchicus*, prepares for diapause primarily during the fifth and final juvenile stage (C5). However, *C. finmarchicus* is not obligated to enter diapause, and some individuals skip diapause, molt immediately into adults, and become reproductively mature. Despite their ecological importance, strikingly little is known about developmental regulation in *C. finmarchicus* or other members of the family Calanidae. Diapause physiology is difficult to study directly in *Calanus* spp. because researchers cannot reliably induce the diapause state under laboratory conditions. Thus, we are currently ignorant of the intrinsic and extrinsic factors that influence the “choice” to enter diapause or to molt directly into adulthood in *C. finmarchicus*, and this ignorance limits our ability to predict population dynamics beyond a single generation.

**Figure 1:**
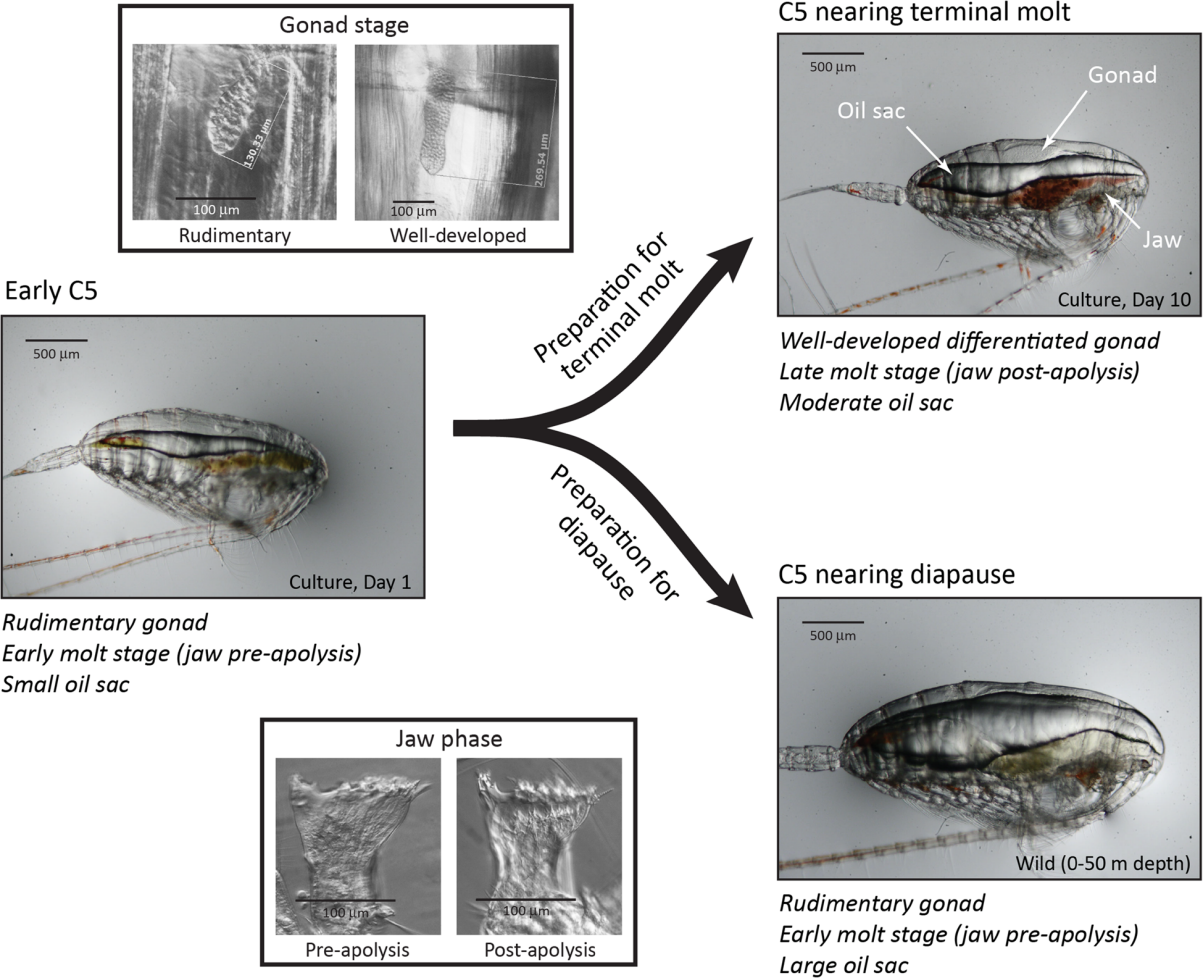
**Morphological changes associated with preparation for diapause or preparation for the terminal molt in** ***Calanus finmarchicus.*** As copepodids progress through the C5 stage toward the terminal molt, the mandibular cuticle separates from the epidermis (apolysis), the gonad develops (grows and changes cell structure) and differentiates (allowing gender to be identified), and the oil sac increases to a moderate size. As copepods progress toward diapause, the oil sac increases to a very large size, but molt stage progression (measured by jaw phase) and gonadal development are arrested.

*Calanus finmarchicus* undergoes dramatic physiological changes during the C5 copepodid stage (Figure 1). The most noticeable of these changes is the accumulation of lipids in an organ called the oil sac. The lipids stored in the oil sac are almost entirely wax esters; much smaller amounts of triglycerides and phospholipids are stored in lipid droplets throughout the body [4]. Lipids accumulated in the oil sac will later be metabolized during fasting by diapausing copepodids, and/or utilized during molting and gonad maturation [5,6], oogenesis [7], or locomotion [8] by adult copepods. The oil sac volume, which can be estimated from analysis of photographs [9], is positively correlated with total lipid content in *C. finmarchicus* C5 copepodids [4]. Gonads appear during the C4 stage [10], but reproductive development accelerates during the C5 stage in copepodids preparing for the terminal molt [7]. Progression toward ecdysis (molting) can be monitored through changes in the mandibular gnathobase during tooth formation [11]; in particular, separation of the cuticle from the epidermis, a processes called apolysis, is a key transition within the arthropod molt cycle that can be detected first in the copepod mandibular gnathobase.

While little is known about regulation of copepod molting, extensive studies in other arthropods (mainly insects and decapods) have shown that molting is regulated by a cascade of hormonal signals (reviewed by [12,13]). Binding of ecdysteroids activates the ecdysone receptor (EcR), which is a member of the nuclear receptor superfamily. EcR forms a complex with another nuclear receptor, the retinoid-x-receptor (RXR) to regulate the transcription of target genes, including several other nuclear receptors (reviewed by [13]). Many studies have characterized EcR and RXR expression during crustacean molt cycles [14-16]; however, few studies have documented expression patterns of other nuclear receptors [17]. While ecdysteroid signaling has not been extensively studied in copepods, Johnson [18] and Hansen et al. [19] found dynamic patterns of ecdysteroid concentration in C5 copepodids from *Calanus* spp. Expression of EcR and other nuclear receptors has not been studied across the molt stage of any copepod, but results from field studies of *C. finmarchicus* are consistent with increased EcR expression in preparation for emergence and molting late in diapause [20,21].

Ontogenetic changes in morphology are often accompanied or even preceded by changes in gene expression, and characterizing these gene expression patterns can help elucidate underlying developmental processes. A few studies have described gene expression patterns associated with progression through the crustacean molt cycle. Genes exhibiting dynamic expression patterns include cuticular proteins [22,23] and nuclear receptors [17]. In *C. finmarchius*, Tarrant et al. [20] suggested that expression of genes associated with lipid synthesis, transport and storage is correlated with lipid accumulation during the C5 stage. Recent studies have employed high-throughput sequencing (RNA-seq) to compare expression among crustacean developmental stages (e.g., [24,25]). Lenz et al. [26] described a *de novo* transcriptome assembled across the entire *C. finmarchicus* developmental cycle (embryos to adults) from which gene expression patterns associated with circadian and peptidergic signaling have been investigated [27,28]; however, these studies have focused on comparisons *between* developmental stages. To gain insight into diapause and maturation processes that happen at the end of juvenile development in *C. finmarchicus,* it is necessary to compare transcriptional profiles *within* the C5 stage.

In this paper, we characterize transcriptional profiles associated with progression through the final copepodid stage of *C. finmarchicus* during preparation for the terminal molt. Our goals were to (1) identify genes associated with lipid storage, reproductive development, and ecdysis during preparation for the terminal molt, (2) characterize changes in the expression of those genes throughout the C5 copepodid stage at high temporal resolution, and (3) understand better the processes and pathways associated with maturation. Our long-term goals are to identify genes associated specifically with preparation for diapause, and to develop markers that can be used to distinguish C5 copepodids that are preparing for the terminal molt and for diapause. With robust molecular markers of these two developmental pathways, new studies can be designed to investigate the factors that influence the “choice” to proceed directly to the terminal molt or to enter diapause. Here, we address the first of these developmental pathways with a transcriptional profiling study of laboratory-reared copepods that are preparing for the terminal molt.

## Results

### Morphometrics, jaw phase, gonads, and stage duration

**Figure 2:**
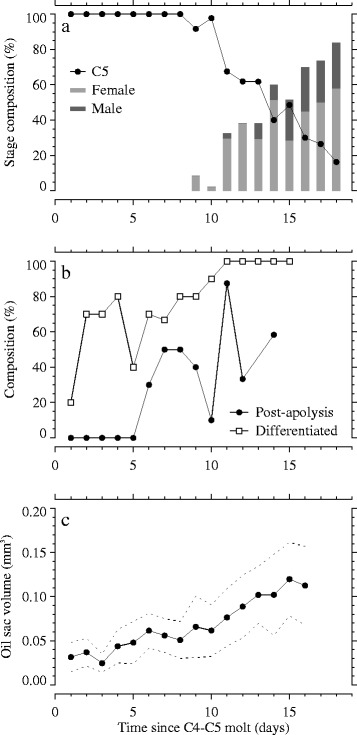
**Time series of developmental stage composition, jaw phase, gonad stage, and oil sac volume. (a)** Daily percentage of *Calanus finmarchicus* C5 copepodids (filled circles) and adults (gray bars) during the time series study (n = 34–44 copepods sampled per day). **(b)** Daily percentage of C5 copepodids with post-apolysis jaw phases (filled circles; n = 8–10 for days 1–12, days 13–15 combined since n = 4 on each of those days, n = 0 for days 16–18) and differentiated gonads (open squares; n = 8–10 for days 1–13, n = 4 for days 14–15, n = 0 for days 16–18). **(c)** Average (filled circles) and standard deviation (dotted lines) of oil sac volume for sampled C5 copepodids (n = 33–43 for days 1–10, n = 12–26 for days 11–16; days 17–18 not plotted since n < 10).

*Calanus finmarchicus* C5 copepodids were sampled from a continuous laboratory culture daily at known times since the C4-C5 (penultimate) molt. Adults first appeared in this time series study 9 days after the penultimate molt, but C5 copepodids were still present on day 18 (Figure 2a). Median C5 stage duration was 13.5 days. Of the 173 copepods that molted into adults during the time series study, 44 (25.4%) were male. In a small sample of C4 copepodids collected prior to the penultimate molt (n = 12), all but one had observable gonads. Of these, 4 had rudimentary gonads, 5 had developing gonads, and 2 were already differentiated (i.e., gender could be determined from well-developed gonads). Not surprisingly then, differentiated gonads were present early in the C5 stage; on nearly all sample days (except days 1 and 5), the majority of C5 copepodids had differentiated gonads (Figure 2b). Of the 98 C5 copepodids sampled with differentiated gonads during the time series study, 44 (44.9%) had developing testes. Apolysis was first observed on day 6, but the percentage of post-apolysis copepodids did not increase monotonically thereafter (Figure 2b), suggesting that the timing of molt stage progression (i.e., within-stage development) is highly variable among individuals. A portion of the variability near the end of the time series was attributable to our including only C5 copepodids in Figure 2b; had we included all individuals that underwent apolysis (C5 and adults), the proportion of post-apolysis *C. finmarchicus* after day 10 would have been higher. In contrast to the variability observed in molt stage progression, average oil sac volume increased steadily 4-fold from day 1 to 16 (Figure 2c).

### Transcriptome assembly

A single library was created from pooled RNA obtained from *C. finmarchicus* C5 copepodids. Illumina-based sequencing produced 93 million 100-bp paired-end reads. After quality trimming, 82.3 million paired-end reads and 10 million unpaired left reads were retained and assembled. The assembled transcriptome contained 124,157 Trinity components and 241,140 Trinity transcripts; multiple transcripts were frequently associated with a single component. The mean transcript length was 667 bp and the N50 (weighted median) was 988 bp. The assembly was qualitatively similar to a recently published *de novo* transcriptome assembled from multiple *C. finmarchicus* life stages (206,041 sequences, average length 997 bp, [26]). The larger average sequence length can be partially attributed to a difference in the minimum sequence length chosen for the two studies (200 bp in the present study, 300 bp in the Lenz et al. [26] study).

### Transcriptome-wide analysis of differential gene expression

**Figure 3:**
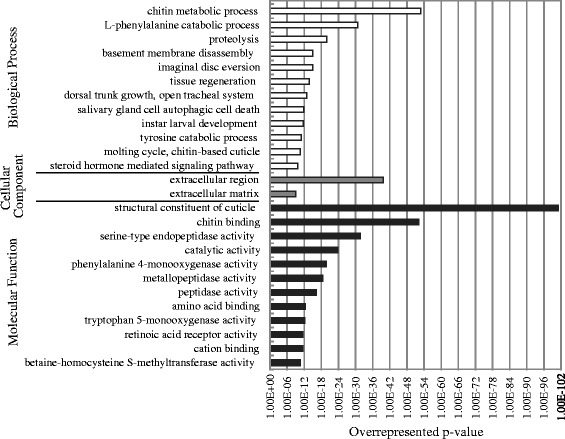
**Gene ontology (GO) terms most strongly enriched (lowest p-value) in the set of differentially expressed genes.** The complete set of statistically overrepresented terms is given in Additional file 3: Table S3.

Transcriptional profiling was conducted on samples collected on days 3 and 10 after the penultimate molt. 22,848 transcripts, corresponding to 7470 Trinity components were differentially expressed between these two days (Additional file 1: Table S1). Of these, one-third (7560 transcripts) returned positive blastx hits based on their similarity to other genes in the NCBI nonredundant (nr) database, and 28% (6479 transcripts) were also associated with gene ontology (GO) terms (Additional file 2: Table S2). The GO terms most strongly enriched in the differentially expressed genes included terms related to remodeling of the cuticle (e.g., chitin metabolic process; structural constituent of cuticle), proteolysis (e.g., tyrosine catabolic process, peptidase activity), and hormonal signaling (e.g., steroid hormone mediated signaling pathway, retinoic acid receptor activity). The categories with the strongest enrichment in the differentially expressed genes are shown in Figure 3; complete results are given in Additional file 3: Table S3.

Of the 100 genes showing the strongest differential expression (lowest p-value), all were expressed at higher levels on day 10. Of these, 58 had no blast hit or only matched predicted proteins with no associated GO terms. A total of 30 genes could be grouped into a small number of categories: myosin heavy chain (14), structural components of cuticle (8), chitin-binding (4), and carbohydrate-binding groups (4). Decapod crustaceans undergo muscular atrophy in preparation for molting; however in American lobster (*Homarus americanus*), this is accomplished with relatively small changes in myosin expression [29]. The very large changes observed in our study may indicate a distinct mechanism of molt-associated muscle remodeling in *C. finmarchicus.* The remaining 12 strongly up-regulated genes fell into 10 categories, including a Torso-like sequence that we selected for detailed expression profiling (see below). Of the genes showing higher expression on day 3, 76 of the 100 genes with the lowest p-values had no blast hit or only matched predicted proteins with no associated GO terms. Among the 24 annotated genes, two were cuticle proteins, and the remaining genes were diverse, each associated with different GO terms. One of these genes was a fatty acid elongase that was similar to a gene we have measured previously (ELOV) and chose for more detailed expression profiling (see below).

Twelve genes were selected for detailed expression profiling in the time series study (Table 1) based on their (1) differential expression in the Illumina study, (2) expression patterns in our previous studies, and/or (3) likely role in reproduction or endocrine regulation of molting. Among these, ELOV (elongation of very long-chain fatty acids) and FABP (fatty acid binding protein) are presumed to function in lipid synthesis and transport; these genes also exhibit high expression in active C5 copepodids with small oil sacs [20]. Vtg is part of the vitellogenin family of lipoproteins that includes major egg yolk proteins (also called apolipocrustaceins in crustaceans) as well as proteins that play non-reproductive roles [30,31]. Fem-1 is an ankyrin domain-containing protein that is necessary for spermatogenesis in *C. elegans* [32]. Fem-1 function is poorly understood in other animal groups, although a Fem-1 homolog has also been suggested to play a role in mammalian prostate development [33]. Torso-like regulates embryonic patterning, developmental timing, body size, and ecdysone secretion [34,35]. ERR (estrogen-related receptor), EcR (ecdysone receptor), FTZ-F1 (FTZ factor 1), HR3, HR38a, HR38b, and HR78 (all named as hormone receptors, but without known ligands) are members of the nuclear receptor family of transcription factors. Nuclear receptors were preferentially selected for qPCR because genes in this family are known to regulate arthropod molting, and commonly show dynamic expression profiles [13,17]. Thus, we considered it likely that genes exhibiting modest differences in expression between the two time points sampled via Illumina might exhibit large changes at other times during the molt stage. The EcR sequence was previously described [20], while the other nuclear receptors were annotated according to a likelihood-based phylogenetic analysis that indicated strong support for grouping with homologs from *Drosophila* and *Daphnia* (Additional file 4: Figure S1). HR38a and HR38b appear to have resulted from a duplication of an ancestral HR38 sometime after the divergence of the copepod and cladoceran lineages (Additional file 4: Figure S1).

**Table 1:** **Sequences (Trinity “components”) identified in the Illumina study and profiled using qPCR**

		**Illumina**			**qPCR**	
**Name**	**Component, accession**	**Log FC**	**Log CPM**	**FDR**	**Log FC**	**Range FC**
FABP	comp254629_c0 ES387222	−2.24	8.57	4.55E-09	−3.45	15.6
ELOV	comp267151_c1 ES387246	−4.72	9.31	4.36E-16	−4.74	30.0
HR78	comp265637_c0 KJ026941	−2.06	0.79	1.02E-04	−1.26	12.8
ERR	comp273411_c0 KJ026943	−2.17	4.89	1.14E-08	−1.84	5.3
HR3	comp264460_c0 KJ026938	3.21	4.48	2.59E-14	3.84	64.2
HR38a	comp270260_c0 KJ026939	2.65	3.63	8.58E-12	2.25	5.9
Vtg	comp274635_c1 KJ026944	3.20	8.14	3.45E-10	1.15	597.9
Torso-like	comp266966_c0 KJ026945	9.26	7.40	1.19e-84	8.24	1024.2
FTZ-F1	comp268775_c0 KJ026942	3.25	5.97	3.05E-16	4.25	32.0
HR38b	comp267178_c0 KJ026940	2.26	4.27	6.58E-10	1.07	7.8
EcR	comp263662_c0 EF583877	1.10	3.13	0.39	1.06	3.4
Fem1	comp269877_c0 KJ026947	4.43	7.08	1.67E-17	1.33	7.6

Log_2_-transformed fold change (FC), log_2_-transformed counts per million (CPM), and false discovery rate (FDR) refer to the Illumina study comparing expression between 3 and 10 days after the penultimate molt. Log_2_-transformed average FC between days 3 and 10 also reported for the qPCR study, as well as the untransformed FC between the minimum and maximum qPCR expression values observed throughout the C5 copepodid stage. Log-transformed FC is reported as a positive number for genes exhibiting greater expression later in the molt cycle (day 10) and a negative number for genes exhibiting greater expression earlier in the molt cycle (day 3).

Some of the genes (but not all) chosen for qPCR analysis were associated with statistically overrepresented GO terms: metal ion binding (ERR, FTZ-F1), retinoic acid receptor activity (HR38a, HR38b), steroid hormone receptor activity (HR38a, HR38b, EcR), retinoic acid receptor signaling pathway (HR38a, HR38b), steroid hormone mediated signaling pathway (HR38a, HR38b, EcR), and nuclear euchromatin (EcR). While GO enrichment analysis can be useful in identifying functionally related groups of co-expressed genes, automatic annotation is not without limitations, particularly for non-model organisms [36]. Some of these limitations can be seen in the above assignments. For example, all of the nuclear receptors that we profiled contain a DNA-binding domain that would be expected to form complexes with zinc ions; however, only a subset of the receptors was assigned with the “metal ion-binding” term.

### Detailed expression profiling

**Figure 4:**
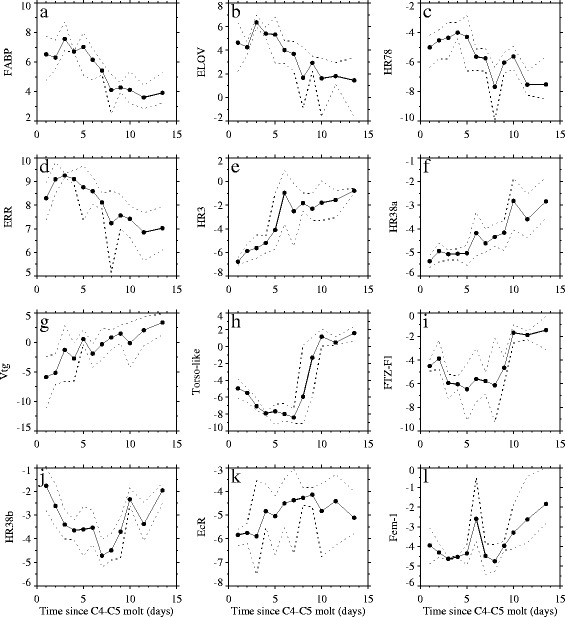
**Average (filled circles) and minimum and maximum (dotted lines) of** ***Calanus finmarchicus*** **C5 copepodid gene expression measured via qPCR during the time series study.** Each of the subpanels **(a-l)** represents the expression profile for an individual gene, which is specified on the y-axis. Average, minimum, and maximum values were computed for n = 3 samples per day for days 1–10; days 11–12 and 13–14 were combined to obtain n = 3 samples for these combined 2-day periods (note that each sample consisted of 3 homogenized copepods). Expression was normalized by 4 housekeeping genes and log_2_ transformed. Negative numbers indicate lower expression than the geometric mean expression of the 4 housekeeping genes (see Methods for additional detail).

The Illumina and qPCR results showed excellent agreement; each of the genes measured by both methods showed similar changes in expression between days 3 and 10 (r^2^ = 0.894, p < 0.0001 for correlation of log-transformed fold changes between days 3 and 10 from the Illumina and qPCR studies; Table 1). Expression of FABP and ELOV peaked on day 3 and decreased thereafter by an average factor of 16 and 30, respectively (Figure 4a,b). Like FABP and ELOV, expression of HR78 and ERR also peaked early in the molt cycle and decreased steadily over time (Figure 4c,d). In contrast, HR3, HR38a, Vtg, and Torso-like each exhibited increasing trends in expression over time (Figure 4e-h). While expression of HR3, HR38a, and Vtg increased fairly monotonically over time, Torso-like expression actually decreased over the first 7 days, increased dramatically by a factor of 768 between days 7 and 10, and remained high after day 10 (Figure 4h). FTZ-F1 and HR38b exhibited U-shaped expression patterns with minimum values observed during the middle of the molt cycle (Figure 4i,j). EcR was not differentially expressed within the Illumina study, and while variability among replicates was very high, there was a weak, inverse U-shaped, average expression pattern with maximum values observed during the middle of the molt cycle (Figure 4k). Finally, expression of Fem-1 showed a more complicated pattern of expression, remaining nearly constant over the first 8 days except for a transient peak in expression on day 6 and an increasing trend after day 8 (Figure 4l).

### Gene expression in copepods of known molt phase

**Figure 5:**
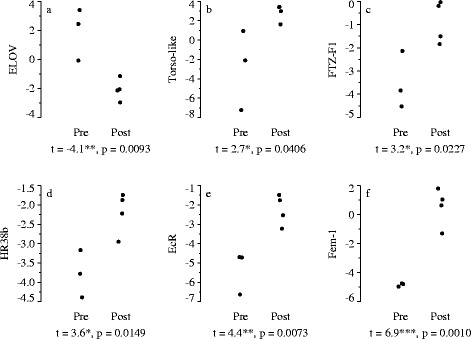
**Gene expression of pre- and post-apolysis** ***Calanus finmarchicus*** **C5 copepodids measured via qPCR 8 and 10 days after the penultimate molt.** Each of the subpanels **(a-f)** indicates the expression level of an individual gene, which is specified on the y-axis. Data consist of n = 3 samples for the pre-apolysis group and n = 4 samples for the post-apolysis group (each sample consisted of 3 homogenized copepods of known molt stage). Expression was normalized by 4 housekeeping genes and log_2_ transformed. Negative numbers indicate lower expression than the geometric mean expression of the 4 housekeeping genes (see Methods for additional detail). Results shown for only those genes with significantly different expression between pre- and post-apolysis (Student’s t-test, p < 0.05).

In animals of known molt stage, expression profiles were broadly consistent with those observed between the middle and late periods of the molt cycle during the time series study (Figure 5). ELOV expression decreased between pre- and post-apolysis samples (p = 0.0093; Figure 5a) whereas expression of Torso-like, FTZ-F1, HR38b, EcR, and Fem-1 increased between pre- and post-apolysis samples (p < 0.05; Figure 5b-f). Although variability among replicates was high and temporal patterns in Fem-1 and EcR were weak during the time series study (Figure 4k,l), these two genes exhibited the strongest differentiation between pre- and post-apolysis samples of all the examined genes (Fem-1: t = 6.9, p = 0.0010; EcR: t = 4.4, p = 0.0073). These results suggest that expression of Fem-1 and EcR is more closely synchronized with the molt cycle than with age within stage, and that variability in the timing of progression through the molt cycle (Figure 2b) caused high variability among replicates during the time series study of Fem-1 and EcR expression.

### Multivariate analysis of gene expression

**Figure 6:**
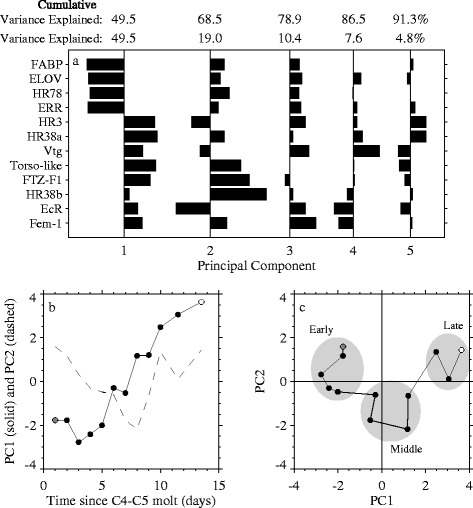
**Multivariate analysis distinguishes three periods of gene expression within the molt cycle.** Results of principal component (PC) analysis of gene expression data from the time series study: **(a)** eigenvectors for the first 5 PCs, **(b)** time series of average PC scores for the first (filled circles) and second (dashed line) PC, and **(c)** scatterplot of average PC scores for the first 2 PCs. Gray-filled and open circles in **(b)** and **(c)** indicate the start and end of the time series, respectively. Gray ellipses in **(c)** indicate early (days 1–5), middle (days 6–9), and late (days 10–14) periods.

Principal component analysis (PCA) was conducted on expression of the 12 normalized genes for 36 samples (approximately 3 replicates per day in the time series study) to better understand overall patterns of expression of this set of genes (Figure 6). The first principal component explained half of the observed variability and captured the dominant linear trends with time for many of the genes: expression of HR3, HR38a, and Torso-like increased over time, while expression of HR78, FABP, ELOV, and ERR decreased over time. The second principal component accounted for an additional 19% of the variability, and captured a U-shaped pattern over time observed in the expression of FTZF-1, HR38b, and EcR (where EcR has a roughly inverted U-shape). The average daily values of the first two principal component scores clustered into 3 time periods: early (days 1–5), middle (days 6–9) and late (days 10–14) (Figure 6c). These time periods, derived from the gene expression data, were consistent with our morphological observations. All C5 copepodids sampled during the early period had pre-apolysis jaws (Figure 2b), indicating they were in the early phase of the molt cycle. The middle period was characterized by the appearance of post-apolysis jaws (Figure 2b). Adults had only just begun to appear at the onset of the late period (Figure 2a), and during this time, nearly all C5 copepodids had differentiated gonads (Figure 2b).

### Gene expression marker of late progression toward the terminal molt

**Figure 7:**
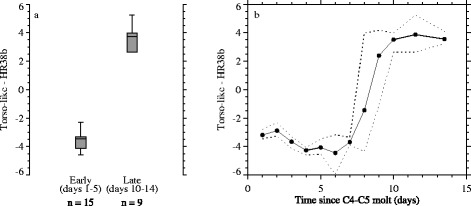
**High Torso-like to HR38b expression indicates late molt stage. (a)** Log_2_-transformed ratio of Torso-like to HR38b expression during the early (days 1–5) and late (days 10–14) periods of the time series study. **(b)** Average (filled circles) and minimum and maximum (dotted lines) of log_2_-transformed Torso-like to HR38b expression ratio.

Determining within-stage progress toward the terminal molt was of particular interest to us, so we sought to develop a gene expression indicator that strongly discriminated between the early (days 1–5) and late periods (days 10–14) within the molt cycle. The expression ratio of all possible pairs of un-normalized genes from the time-series study (n = 12 genes) were first calculated and log_2_ transformed. Then the Student’s t-statistic comparing the ratio between the early period (n = 15 samples) and late period (n = 9 samples) was calculated for each gene pair. The gene pair with the highest t-statistic was considered the best discriminator between the early and late periods. Of the 66 possible gene expression ratios, the ratio of Torso-like to HR38b had, by far, the highest t-statistic (t = 24.1, p < 0.0001; note that p < α, where α = 0.05/66 = 0.00076, the Bonferroni adjusted cutoff value; Figure 7a). During the early period, HR38b expression was an average 12.2 times higher than that of Torso-like, and during the late period, Torso-like expression was an average 12.5 times higher than that of HR38b (Figure 7a). The middle period (days 6–9) was characterized by a dramatic change in the ratio (Figure 7b). While Torso-like alone provides good discrimination between the early and late periods (Figure 4h), the ratio of Torso-like to HR38b provides better discrimination because the ratio is less variable during the early period when expression levels of both genes decrease simultaneously.

## Discussion

The lipids stored in the *C. finmarchicus* oil sac provide energy to support basal metabolism during diapause, as well as molting and reproduction. The present study provides, to our knowledge, the first dynamic profile of oil sac volume in *Calanus spp.* C5 copepodids progressing toward the terminal molt. Oil sac volume is strongly correlated with wax ester content in *C. finmarchicus* C5 copepodids [4]. While triglycerides are typically present in relatively small amounts, they appear to serve as a labile energy source that is utilized both during short periods of starvation and during egg production [4,37]. Thus, it will be of interest to profile wax ester and triglyceride content in future studies of *C. finmarchicus* development, as well as to identify molecular markers associated with synthesis and utilization of each lipid type. During our study, oil sac volume increased over time, presumably to fuel molting, reproduction and adult metabolic needs [5-7], and genes associated with lipid accumulation (FABP and ELOV) peaked on day 3 and decreased afterward. In a field study of *C. finmarchicus* C5 copepodids, we showed that expression of FABP and ELOV was highest in active animals with small oil sacs [20]. If these copepods were early developing C5 copepodids, then their high FABP and ELOV expression is consistent with the high FABP and ELOV expression observed in the early developing C5 copepodids from our culture study. In this previous study [20], we assumed that changes in FABP and ELOV expression were associated with diapause preparation, but the present study shows that changes in the expression of these genes are more generally associated with lipid accumulation, which is necessary both for diapause and progression toward the terminal molt. ERR expression also peaked on day 3 and decreased afterward. While the specific function of this nuclear receptor in *C. finmarchicus* is unknown, ERR is an important regulator of energetic homeostasis and lipid metabolism [38,39].

During progression through the C5 stage, the proportion of copepodids with differentiated gonads increased. While nearly half (44%) of the copepodids with differentiated gonads possessed testes, only 25% of the emerged adults were male. Differences in the sex ratio of wild copepod populations are frequently attributed to differential mortality (e.g., [40]), but mortality was extremely low in the culture study. Sex ratio in *Calanus* spp. can be influenced by food availability, food quality, population density and other environmental factors, and cultured populations are usually biased toward females ([41] and references therein). Because we sampled most of the copepodids prior to the terminal molt, we cannot be sure what proportion of the population would have eventually emerged as adults. One possibility is that males develop more slowly, which is weakly supported by our data (Figure 2a). A second intriguing possibility is that sex change accounts for the observed difference in sex ratio, as has been previously suggested for *C. finmarchicus* (reviewed by [42]).

We profiled the expression of three genes, Vtg, Fem-1, and Torso-like, each of which is associated with reproductive development in other animals. We identified several Vtg-like transcripts, most of which did not exhibit differential expression between days 3 and 10. The Vtg transcript profiled in this study increased over time in the C5 stage, but decreased in both male and female adults (adult expression not shown). This differs from Vtg expression profiles described in other copepods in which expression is highest in adult females where it plays a role in egg yolk production [43,44]. Closely-related genes (e.g., Crossveinless d) serve non-reproductive developmental roles in insects [31]. While we do not know the specific role of the Vtg-like gene we have profiled in *C. finmarchicus,* we consider it likely that it serves a developmental role unrelated to egg yolk production. Fem-1 expression exhibited high variability among replicates, weak temporal patterns, and a strong increase after apolysis. This gene helps to regulate spermatogenesis in *C. elegans* [32]*,* so the weak temporal variability observed within the culture may be due to sexually dimorphic expression. The strong discrimination between pre- and post-apolysis animals of similar time in stage may indicate a transitory peak in expression not resolved in the time series study. Torso-like has been studied most extensively in *Drosophila,* in which it is transmitted from the ovary to the oocyte to regulate development of the head and tail regions of the resulting embryo; however, this role of Torso-like embryonic patterning is likely a derived function restricted to a few insect lineages [45]. While the function of Torso-like is unknown in *C. finmarchicus*, the large dynamic range in expression with a maximum in post-apolysis animals is consistent with a conserved role in regulation of molting hormones, as has been shown in insects: [34,35].

EcR did not exhibit differential expression between days 3 and 10 in the Illumina study. In the time series study, EcR expression was greatest during the middle period, but temporal trends were weak. This weak temporal pattern can be partially attributed to asynchrony within the molt stage obscuring developmental changes, as evidenced by the strongly differentiated expression of EcR before and after apolysis (Figure 5e). In insects, EcR regulates expression of other nuclear receptors including HR78 and HR3, and HR3 in turn induces expression of FTZ-F1 (reviewed by [13]). In *C. finmarchicus,* several other nuclear receptors (HR38b, HR3, FTZ-F1) exhibited dynamic profiles during the time series study. The dynamic expression profiles of these genes may by driven primarily by changes in ecdysteroid concentrations rather than changes in the expression of EcR. In insects, HR78 and HR38 both oppose EcR signaling [46,47]. In our study, HR78 expression was highest early in the molt cycle, with peak expression on day 4. This preceded the peak in EcR expression observed on day 9, which may have prevented premature activation of ecdysis pathways. Of the two HR38 genes in *C. finmarchicus*, HR38a increased over time, and HR38b exhibited a U-shaped pattern similar to FTZ-F1 that was broadly in opposition to EcR. Expression patterns of HR38b and FTZ-F1 were closely associated with molt progression, exhibiting a strong increase after apolysis (Figure 5c,d). HR3 expression generally increased over time and showed a transient peak on day 7. Among copepods collected on days 8 and 10, HR3 expression was highly variable prior to apolysis (not shown), which is consistent with an ephemeral peak in HR3 expression shortly before apolysis.

We identified several genes that we expect will be useful for identifying wild C5 copepodids that are in the late stages of preparation for the terminal molt. The copepods used in this study were all taken from a long-term continuous culture in which the animals are reared at a constant temperature, with a reliable food supply, without any exposure to predators, and at higher densities than they generally occur in nature. For reasons that are not known, *Calanus* spp. does not undergo diapause in culture, and we do not understand in what other ways the physiology and corresponding gene expression of cultured and wild C5 copepodids differ. Therefore, it will be necessary to compare the gene expression profiles observed in our culture study with wild populations that are preparing for the terminal molt. We anticipate that Torso-like and HR38b, whose ratio changes dramatically during C5 development, may prove especially useful for identifying copepods nearing the terminal molt. The lipid accumulation hypothesis of diapause initiation [48-50] predicts that copepods that skip diapause and proceed directly to the terminal molt have lower lipid reserves than those preparing for diapause. With a biomarker that predicts late molt stage, the lipid volume of copepods preparing for the terminal molt can be directly examined in the field. Moreover, this biomarker can be used to identify animals in the field that are *not* actively preparing for the terminal molt, of which some will be preparing for diapause. Within this subpopulation of C5s, we anticipate that additional transcriptional profiling and gene expression studies will identify genes directly associated with preparation for diapause during the C5 stage. The development of robust gene expression biomarkers of these two developmental pathways will ultimately enable novel studies of the intrinsic and extrinsic factors that govern diapause initiation and termination in *Calanus finmarchicus*.

## Conclusions

This study provides the first dynamic profiles of gene expression through a copepod molt cycle. In addition to processes of growth and ecdysis that are common to all molt stages, the *C. finmarchicus* C5 stage is also characterized by lipid storage and gonad development. These processes are interrelated in the sense that lipid stores provide energy to fuel ecdysis and reproduction. Broadly, we have observed that FABP and ELOV expression is high early in the C5 stage, and decreases over time as lipids accumulate. Several nuclear receptors exhibited dynamic expression profiles consistent with a signaling cascade triggered by ecdysteroids. We measured, for the first time, gene expression in copepods of known molt stage and identified genes that exhibit dramatic changes in expression during apolysis. These include genes with presumed roles in lipid synthesis, molt regulation and gonad development, which is consistent with a coupling of these processes. Finally, we identified the ratio of Torso-like to HR38b as a biomarker that strongly differentiates between early and late development within the C5 copepodid stage.

## Methods

### Culturing

Copepods were collected from a long-term continuous culture of *C. finmarchicus* maintained at the NTNU/SINTEF SeaLab facility in Trondheim, Norway [19]. Copepods were reared at 8–10°C in 280-l polyester containers through which filtered natural seawater from nearby Trondheimsfjord (collected at 70 m depth) was continuously passed. All copepods were fed a diet of *Dunaliella tertiolecta, Isochrysis galbana,* and *Rhodomonas baltica* at levels > 150 μg C l^−1^. During May 2012, ~1900 stage C4 copepodids were removed from the culture and transferred to 2.5-l containers (40 individuals per container, 48 containers). Every day for 9 consecutive days, each copepod was gently removed with a 17 cm^3^ ladle, transferred to a clear plastic spoon, and examined under a stereomicroscope. If an individual had molted from C4 to C5 during the preceding ~24 hours, it was transferred to a new 2.5-l container and not examined again until its sample date (see below). A total of 34 2.5-l containers were filled with 35–47 (average of 38) C5 copepodids, resulting in 1306 individuals for whom the date of the penultimate (C4 to C5) molt was known to within a day.

### Sampling

Containers with C5 copepodids were assigned randomly to particular sample dates to allow data collection on each day after the penultimate molt, including days 1–18 for a time series study and days 3 and 10 for Illumina-based transcriptional profiling (see below). All animals from a single container with the same penultimate molt date were sampled simultaneously. Individuals were removed from the container with a ladle, staged on a depression slide, photographed alive with a Canon EOS-20D camera attached to a Zeiss Stemi 2000C stereomicroscope, and preserved either in RNAlater (Ambion) for quantitative polymerase chain reaction (qPCR) or transcriptional profiling, or in 10% buffered formalin for assessment of jaw phase or gonad stage. For the time series study, individuals from each container were randomly assigned to one of three analysis types: jaw phase (n = 10 copepods), gonad stage (n = 10 copepods), or qPCR analysis (n = 15 copepods). These sample sizes could not be maintained during the last few days of the time series study when the occurrence of C5 copepodids was low (because many molted into adults prior to sampling), so data from these last days were combined for all analyses. For qPCR and Illumina analyses, replicates of 3 copepods each were preserved in RNAlater.

### Morphometrics, jaw phase, and gonads

Prosome length and width and oil sac volume of each sampled copepod were estimated with measurements obtained from digital photographs of the live animals (after Tarrant et al. [20]). Measured lengths were calibrated with repeated photographs of a stage micrometer taken during sampling. Jaw phase was assessed by extracting the mandible under a stereomicroscope, mounting it in glycerine under a cover slip, and photographing the gnathobase under a Zeiss Axiovert 40 inverted compound microscope outfitted with differential interference contrast optics and a Zeiss AxioCam ICc1 camera. Jaws were classified after Miller et al. [11] and Crain and Miller [51]. Apolysis, the process by which the epidermis separates from the cuticle, is an important milestone in molt preparation; we therefore refer to pre-apolysis copepodids as those with jaws in the post-molt, late post-molt, interphase, or columnar cell tooth-forming phases, and post-apolysis copepodids are those with jaws in any of the other late tooth-forming phases (apolysis, tooth molds, silicified teeth, or extricated teeth). To assess gonad stage, C5 copepodids were stained by passing them through 5 baths of seawater serially diluted with deionized water (100, 75, 50, 25, 12.5% seawater for 20 minutes each), a deionized water bath of 3% Fast Green (20 minutes), 3 baths of deionized water, and 5 baths of increasing ethanol concentration (15 minutes in 15, 40, and 75% ethanol, 37 minutes each in 95 and 100% ethanol); final preservation was in terpineol. Gonads were viewed with an inverted compound microscope and classified according to Crain and Miller [52]. Differentiated gonads are defined as those from which an individual’s gender can be determined (i.e., developing ovaries and testes).

### RNA extraction and library preparation

Total RNA was extracted from copepods using the Aurum Fatty and Fibrous Tissue Kit (Bio-Rad). RNA purity and yield were assessed using a Nanodrop spectrophotometer. For the time series study, on-column DNase digestion was used to remove genomic DNA, and RNA quality was assessed using denaturing agarose gels. For the Illumina study, RNA quality was assessed using a Bioanalyzer. Samples were submitted to the Genomic Services Laboratory at HudsonAlpha (Huntsville, AL), where libraries were synthesized using Illumina TruSeq reagents. Four libraries were constructed from each of days 3 and 10, and each library was prepared using total RNA extracted and pooled from enough copepods to yield at least 4 μg of RNA (3–6 copepods). For transcriptome assembly, an additional library was constructed from equal amounts of RNA pooled from each of these eight samples (2 μg total) along with 2 μg of RNA from wild C5 *C. finmarchicus* collected in Trondheimsfjord, Norway during May 2012*.* RNA from wild copepods was included in the library to facilitate future field-based transcriptional profiling studies.

### Illumina-based sequencing and data analysis

For transcriptome assembly, samples were sequenced with 100 base pair (bp) paired-end reads with a depth equivalent to ½-lane on an Illumina HighSeq2000. For expression profiling, samples were sequenced with 50 bp paired-end reads with a depth equivalent to ¼-lane per sample. Adapter sequences and bases with low quality scores (< phred 20) were removed using Trimmomatic [53] in paired-end mode. After trimming, reads at least 50 bp long were retained for assembly. A transcriptome was assembled *de novo* using Trinity version r2012-06-08 [54] with default parameters. Transcripts were annotated using Blast2Go [55] with a threshold e-value of 10^−6^. Post-assembly analysis was conducted using scripts bundled within the Trinity package. Reads were mapped to the reference transcriptome and quantified using the RSEM software package [56]. Read counts were TMM-normalized (trimmed mean of M-values) to account for differences in library size [57] and then FPKM-normalized (fragments per feature kilobase per million reads mapped) to account for differences in transcript length. Transcripts exhibiting differential expression between the two time points were identified using EdgeR software [58]. Default parameters were used, including a p-value cutoff for false discovery rate (FDR) of 0.001 and a minimum 4-fold change in expression. Statistically enriched gene ontology (GO) terms were identified using the GOseq R Bioconductor package [59].

### Cloning and phylogenetic analysis

Portions of transcripts of interest (672–1313 bp) were amplified by PCR (as in [20]), both to verify the assembled sequence and to provide standards for qPCR. All primers are provided in Additional file 5: Table S4. Amplicons were cloned into pGEM-T Easy (Promega) and sequenced. To improve the provisional annotation of several transcripts within the nuclear receptor superfamily, the cloned *C. finmarchicus* sequences were aligned with the complete nuclear receptor sets from human, *Drosophila melanogaster* and *Daphnia pulex,* excluding the atypically structured nuclear receptors (Family 0). Amino acid sequences were aligned using ClustalW, and maximum likelihood analysis was conducted with RAxML v7.0.4 [60] using the JTT + I + G + F model.

### Quantitative PCR (qPCR)

Complementary DNA (cDNA) was synthesized from 450 ng of total RNA in a 20-μl reaction using the Iscript cDNA synthesis kit (Bio-Rad). The 20-μl reaction was diluted with 30 μl of molecular biology grade water such that each microliter of diluted cDNA corresponded to 9 ng of total RNA. qPCR was performed using SsoFast EvaGreen Supermix (Bio-Rad) on an iCycler iQ Real-Time PCR detection system (Bio-Rad). Primer sequences for ELOV, FABP and EcR were previously described [20], and all other primer sequences are given in Additional file 5: Table S4. Standards were prepared from a serially diluted plasmid containing the amplicon. All samples and standards were run in duplicate wells. The 20-μl qPCR reaction contained 10 μl master mix, 8 μl molecular biology grade water, 1 μl diluted cDNA and 1 μl 10 nM primers. Cycling parameters were: 95°C for 2 min followed by 40 cycles of 95°C for 5 s and 63°C for 10 s. After cycling, the products were subjected to melt curve analysis to ensure that only a single specific product was amplified.

Transcript expression was calculated relative to the standard curve and log_2_-transformed. Expression was further adjusted by subtracting a normalization factor equal to the geometric mean of 4 genes that exhibited relatively constant levels of expression after [61]. Three of these normalizer genes were selected from the Illumina data to have moderate expression levels and a low coefficient of variation among samples (TSG: KJ026946 tumor susceptibility gene 101 protein-like, UBX: KJ026948 UBX domain-containing protein 6-like, and SNP: KJ026949 snurportin 1-like). The fourth normalizer gene (EF-1α: ES414812, similar to elongation factor 1 alpha) has been used in previous qPCR studies of *C. finmarchicus* [19] and exhibited stable expression in our study.

Gene expression was measured with qPCR on days 1–16 of the time series study. Three biological replicates were measured on days 1–10, 2 replicates were measured on days 11, 13, and 16, and 1 replicate on days 12, 14, and 15. Results from days 11–12 and days 13–14 were combined in all figures. Results from days 15 and 16 were excluded from further analysis because those days exceeded the median C5 stage duration of the culture.

Because molt stage progression was not synchronous within the culture (see Results), we used RNAlater-preserved samples collected on days 8 and 10 to simultaneously assess jaw phase and measure gene expression in the same individual C5 copepodids. Mandibles were extracted and examined, and after jaw phase was determined, the rest of the body was grouped with copepods that had similar jaw phases. Each replicate sample for qPCR consisted of total RNA extracted and pooled from 3 copepods with either (1) jaw phases occurring immediately before apolysis (interphase or columnar cell tooth forming phases) or (2) jaw phases occurring after apolysis (apolysis, tooth molds, silicified teeth, or extricated teeth). We analyzed 3 and 4 replicates each for pre- and post-apolysis jaw phases, respectively.

#### Availability of supporting data

The cloned DNA sequences (Genbank accessions KJ026938-KJ026947), raw Illumina sequences, and Transcriptome Shotgun Assembly are archived at NCBI (NCBI BioProject: PRJNA231164 [GenBank:PRJNA231164], Transcriptome Shotgun Assembly DDBJ/EMBL/GenBank Accession GBFB00000000. The version described in this paper is GBFB01000000): Other data sets supporting the results of this article are included within the article and its additional files.

## Additional files

Additional file 1: Table S1. Tab-delimited file containing normalized counts (FPKM) and RSEM statistical analysis of Trinity components differentially expressed between days 3 and 10. Column 1: Comp Name, the name of the differentially expressed Trinity component. Column 2: logFC, the log_2_-transformed fold change in expression; positive numbers indicate higher expression on day 10 of the time series relative to day 3. Column 3: logCPM, log_2_-transformed counts per million. Column 4: p-value, indicating significance of difference in expression between days 3 and 10. Column 5: FDR, false discovery rate. Columns 6-9: normalized expression values for biological replicates collected on day 3 (EC1.genes-EC4.genes). Columns 10-13: normalized expression values for biological replicates collected on day 10 (LC1.genes-LC4.genes). Column 14: # annotated seqs: the number of transcripts (“seqs”) corresponding to a given line (component) that are annotated in Additional file 2: Table S2. Columns 15-22: the blast2GO-based annotation based on the first corresponding sequence present in Additional file 2: Table S2 (if any). Additional file 2: Table S2. Tab-delimited file containing annotation of transcripts with a positive blastx hit (e-value < 10^-6^) that were differentially expressed between days 3 and 10. Column 1: Trinity transcript name. Column 2: Sequence description. Column 3: Sequence length. Column 4: Number of blast hits. Column 5: Lowest e-value of significant blast hit. Column 6: Mean percent similarity. Column 7: Number of associated GO terms. Column 8: List of associated GO terms. Additional file 3: Table S3. Microsoft Excel file containing the complete list of gene ontology (GO) terms that are statistically overrepresented in the set of genes differentially expressed between days 3 and 10. Additional file 4: Figure S1. Maximum likelihood tree of nuclear receptors from human (*Homo sapien*; Hs), *Drosophila melanogaster* (Dm) and *Daphnia pulex* (Dp) with newly-identified *Calanus finmarchicus* (Cf) nuclear receptors shown in red. Nuclear receptors are typically grouped into 6 families (a seventh family with contains genes with atypical structure is not shown), some of which are further divided into subfamilies [62]; these designations are indicated on the right side of the figure. Accession numbers for *C. finmarchicus* sequences are given in Table 1. All other accession numbers are given in Additional file 1 of [63] Tree is unrooted. Values to the right of nodes indicate percent of 1,000 bootstraps. Scale bar at bottom indicates the number of amino acid substitutions per site. Trees were visualized using FigTree v1.1.2 (http://tree.bio.ed.ac.uk/software/figtree/). Additional file 5: Table S4. Word document. Primers used for cloning of plasmid standards and for quantitative PCR.

## Abbreviations

Bp: Base-pair
C4 and C5: Fourth and fifth copepodid stage, respectively
cDNA: Complementary DNA
CPM: Counts per million
DNA: Deoxyribonucleic acid
EcR: Ecdysone receptor
EF-1α: Elongation factor 1 alpha
ELOV: Elongation of very long-chain fatty acids
ERR: Estrogen-related receptor
FABP: Fatty acid binding protein
FDR: False discovery rate
FPKM: Fragments per feature kilobase per million reads mapped
FTZ-F1: Fushi tarazu transcription factor 1
GO: Gene ontology
HR: Hormone receptor (e.g., HR38: hormone receptor 38)
nr: Non-redundant (database)
PC(A): Principle component (analysis)
PCR: Polymerase chain reaction
qPCR: Quantitative PCR
RNA: Ribonucleic acid
RXR: Retinoid-x-receptor
SNP: Snurportin
TMM: Trimmed mean of M-values
TSG: Tumor suppressor gene
Vtg: Vitellogenin
