# Supplementary figures and images for: Transcriptional profiling of reproductive development, lipid storage and molting throughout the last juvenile stage of the marine copepod Calanus finmarchicus
Source: Front Zool. 2014 Dec 16;11:91. doi: 10.1186/s12983-014-0091-8 (PMC4285635; doi:10.1186/s12983-014-0091-8)

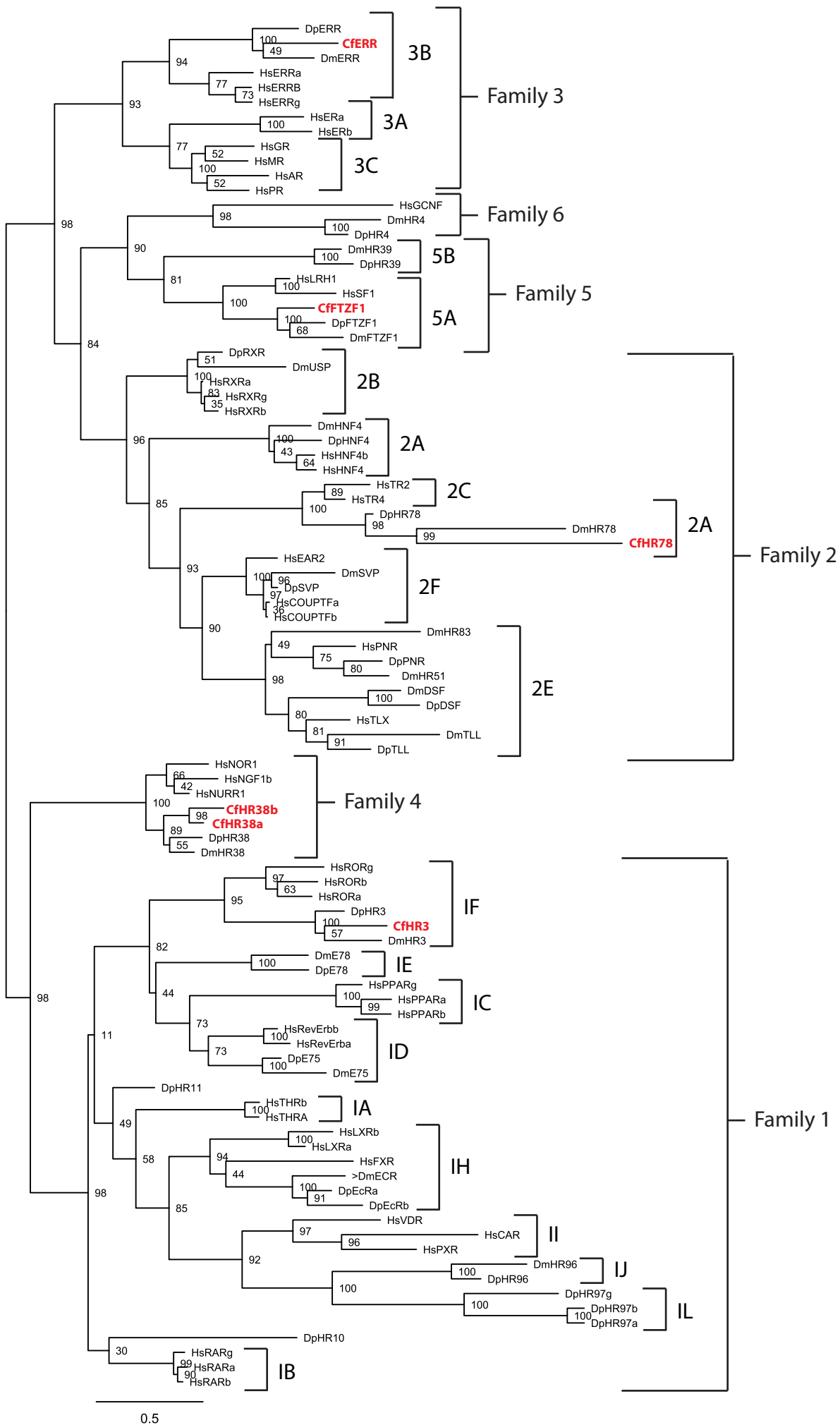

Supplement: Additional file 4: Figure S1. — Maximum likelihood tree of nuclear receptors from human (Homo sapien; Hs), Drosophila melanogaster (Dm) and Daphnia pulex (Dp) with newly-identified Calanus finmarchicus (Cf) nuclear receptors shown in red. Nuclear receptors are typically grouped into 6 families (a seventh family with contains genes with atypical structure is not shown), some of which are further divided into subfamilies [62]; these designations are indicated on the right side of the figure. Accession numbers for C. finmarchicus sequences are given in Table 1. All other accession numbers are given in Additional file 1 of [63] Tree is unrooted. Values to the right of nodes indicate percent of 1,000 bootstraps. Scale bar at bottom indicates the number of amino acid substitutions per site. Trees were visualized using FigTree v1.1.2 (http://tree.bio.ed.ac.uk/software/figtree/). [file 12983_2014_91_MOESM4_ESM.pdf]
