# Supplementary material for: Transcriptional profiling of reproductive development, lipid storage and molting throughout the last juvenile stage of the marine copepod Calanus finmarchicus
Source: Front Zool. 2014 Dec 16;11:91. doi: 10.1186/s12983-014-0091-8 (PMC4285635; doi:10.1186/s12983-014-0091-8)
Supplement: Additional file 5: Table S4. — Word document. Primers used for cloning of plasmid standards and for quantitative PCR. [file 12983_2014_91_MOESM5_ESM.docx]

Table S4: Primers used for cloning of plasmid standards and for quantitative PCR.

| Gene | Cloning primers (fragment size, nt) | qPCR primers (amplicon size, nt) |
| --- | --- | --- |
| HR38a | F:5’- CCCCAGTCTCCATCACTTCTATGC-3’ (779)  R:5’- GTAAGGGCTGCCAAACATGAAAAG-3’ | F:5’- GGAGCTGCTCTTCCAATCAGC-3’ (131)  R:5’- AGGTGGATCGGGCTTGATTTC-3’ |
| HR3 | F:5’- CTAGCAGACCCACCCCACCC-3’ (672)  R:5’-GCTCCCGCTGCAGTGAGTTG-3’ | F:5’- TTTGTGCCGCCCAGAGATTGAC-3’ (108)  R:5’- CGGCCAACTCAAAACTTCCAGC-3’ |
| FTZ-F1 | F:5’- TTCAAACTGCCACGCTCCCTTG-3’ (737)  R:5’-TCGGCAGTTGTGTTTCGTCAGG-3’ | F:5’- AGAGCATCCAGTGGCAGATCAG-3’ (111)  R:5’-AAGATGCCGTCGACACCAATCC-3’ |
| HR38b | F:5’- AAAGCTCTGCATCCCATCATCTTC-3’ (1092)  R:5’- GAGAAATCCGAGCAGCTTCAAGAG-3’ | F:5’- CGGTTTCAGAAGTGCCTTGC-3’ (101)  R:5’- TTGCTCCGAGGTTTTGTTGG-3’ |
| ERR | F:5’-AGCTGTCTTGTGTGCGGAGATG-3’ (689)  R:5’- CGCTCCATCCGTTCCACAATCAAC-3’ | F:5’-TAGGCGCTATCCCATGGAAAC-3’ (121)  R:5’-CAGCGATACCAGTTGCTCAGG-3’ |
| HR78 | F:5’- TAAGAGTTCCAGCCAAAGCAGAGG-3’ (923)  R:5’- TTTCAAAGCCTGAGAAGGTTGGTC-3’ | F:5’- TACCGCAACGAAACACCAACC-3’ (141)  R:5’- TCAAGAGGTCCACCTGGCTTG-3’ |
| Vtg | F:5’- ACCCCTGTGGACACATTCTCAAGG-3’ (999)  R:5’- TCCCTTGGGAAACCACATGTTGAG-3’ | F:5’- TGCTTGGACACAGCCAAACAG-3’ (113)  R:5’- TTGGGAAACCACATGTTGAGC-3’ |
| Torso-like | F: 5’-GAGGCACCAGACAAACAAACATGG-3’ (1123)  R:5’-TTCCTCCTCATTGCTCTCATCTGG-3’ | F:5’-TCTGTCAGTGCGCGTAGTTCC-3’ (109)  R:5’-CAGAGGTTGCTTGACCTGACG-3’ |
| Fem1 | F:5’- AACAGAAGTGAGCTGGACGCCTTG-3’ (958)  R:5’- TTTCCAGCACTTCAGGCACCTCTC-3’ | F:5’- CAACATTTTGGGCTGGTTTGG-3’ (110)  R:5’- TTCCATGCATGTTCCACCAAC-3’ |
| SNP | F:5’-CAGGGTCATTCAATGTCACCAATG-3’ (796)  R:5’-GTCTTCCCAGGCATGTAGTGAACG-3’ | F:5’-GAACTGGCTGCTTCTCCTGTG -3’ (98)  R:5’-TCACTTGCTTCCCACTCTTGC-3’ |
| TSG | F:5’-AGGGACAGCATCAAGCATCAGTTC-3’ (434)  R:5’-TGAGGAACACCTCCACATCAATGG-3’ | F:5’-GAACACATCAAGGCAAGCATCC (86)  R:5’-TCAGCTTGGTTCTGGCTGAAG-3’ |
| EF-1α | F:5’-GGCATTGACAAGAGGACCATCG-3’ (1194)  R:5’-CCTCATGTCACGGACAGCGAAC-3’ | F:5’-CCTCCGACTCCAAGAACAAGC-3’ (127)  R:5’-ATATGGGCGGTGTGACAATCC-3’ |
| UBX | F:5’-AGTCTGGCTGCGAAGGGAGTCTAC-3’ (477)  R:5’- AAGTCCTCGGTCCAGTTCAGCAG -3’ | F:5’-AGGGTCCTGATGGCCAAATG-3’ (128)  R:5’-TCCTCGGTCCAGTTCAGCAG-3’ |
